# Biological functions of m^6^A methyltransferases

**DOI:** 10.1186/s13578-020-00513-0

**Published:** 2021-01-11

**Authors:** Jianzhong Gu, Yu Zhan, Lvjia Zhuo, Qin Zhang, Guohua Li, Qiujie Li, Shasha Qi, Jinyu Zhu, Qun Lv, Yingying Shen, Yong Guo, Shuiping Liu, Tian Xie, Xinbing Sui

**Affiliations:** 1 College of Pharmacy, School of Medicine, Hangzhou Normal University, Hangzhou, 311121 Zhejiang China; 2 Department of Oncology, The First Affiliated Hospital of Zhejiang Chinese Medical University, 54 Youdian Road, Hangzhou, 310006 Zhejiang China; 3 Key Laboratory of Elemene Class Anti-Cancer Chinese Medicines, Engineering Laboratory of Development and Application of Traditional Chinese Medicines, Collaborative Innovation Center of Traditional Chinese Medicines of Zhejiang Province, Hangzhou Normal University, Hangzhou, 311121 Zhejiang China; 4 Department of Respiratory medicine, the Affiliated Hospital of Hangzhou Normal University, School of Medicine, Hangzhou Normal University, Hangzhou, 310015 Zhejiang China

**Keywords:** M^6^A methyltransferase, Growth and development, Metabolism, Infection and immunity, Tumour progression

## Abstract

M^6^A methyltransferases, acting as a writer in N6-methyladenosine, have attracted wide attention due to their dynamic regulation of life processes. In this review, we first briefly introduce the individual components of m^6^A methyltransferases and explain their close connections to each other. Then, we concentrate on the extensive biological functions of m^6^A methyltransferases, which include cell growth, nerve development, osteogenic differentiation, metabolism, cardiovascular system homeostasis, infection and immunity, and tumour progression. We summarize the currently unresolved problems in this research field and propose expectations for m^6^A methyltransferases as novel targets for preventive and curative strategies for disease treatment in the future.

## Background

N6-methyladenosine (m^6^A) is acknowledged as one of the most ubiquitous and abundant mRNA methylation modifications that occurs in eukaryotes. It was discovered in 1974, when Desrosiers and colleagues studied the methylation status of hepatoma cells mRNA using the polyadenosinic acid (PolyA) structure in eukaryotes [1]. M^6^A is an epigenetic mark, a heritable change driven by chemical modifications that alters gene expression without changing the nucleotide sequence [2]. M^6^A methylation modification is a reversible modification, which is methylated by m^6^A methyltransferases (writers), demethylated by m^6^A demethylases (erasers) and also recognized by m^6^A binding proteins (readers), participating in a series of biological processes [3, 4]. This review focuses on the writers, i.e. m6A methyltransferases.

M^6^A methyltransferases form a stable complex catalysing the methylation of RNA [5]. This complex consists of two core components, methyltransferase-like 3 protein (METTL3) and methyltransferase-like 14 protein (METTL14), and other accessory regulatory subunits, such as Wilm’s tumour-1-associated protein (WTAP)/(Fl(2)d) [6], KIAA1429 (Virilizer), Hakai, RBM15, METTL16, which need to be further explored. METTL3 (initially called MT-A70) was first discovered in 1997 as a major component in an ~ 200-kDa complex isolated from a mammalian cell nuclear extract that exhibited methyltransferase activity, marking a significant breakthrough in m^6^A methyltransferase research [7, 8]. Although METTL14 shares 43% homology with METTL3 [9], it does not have catalytic activity, as indicated through crystal structure studies [10]. METTL14 provides an RNA-binding scaffold, facilitates allosteric activation and promotes the catalytic activity of METTL3 [3]. These two proteins form a stable heterodimer core complex with a 1:1 stoichiometry and function synergistically both in vitro and in vivo [8, 11]. WTAP localizes the m^6^A methyltransferase complex to nuclear speckle targets enriched with pre-mRNA and increases its catalytic activity [6, 12]. In mammalian cells, KIAA1429, Hakai and RBM15 are components associated with WTAP that recognize candidate methylation sites and perform precise post-transcriptional regulation [13], while ZC3H13 acts as a bridge between RBM15 and WTAP [14]. METTL16 is an effective component that has recently been discovered, with only partial cellular localization abilities and RNA-binding preferences worthy of further exploration [15]. Moreover, these m^6^A methyltransferases in mammals have homologues in *Saccharomyces cerevisiae*, *Drosophila*, zebrafish, *Arabidopsis thaliana* and others (described in more detail below), which play similar roles in life processes.

### Growth and development

**Fig. 1:**
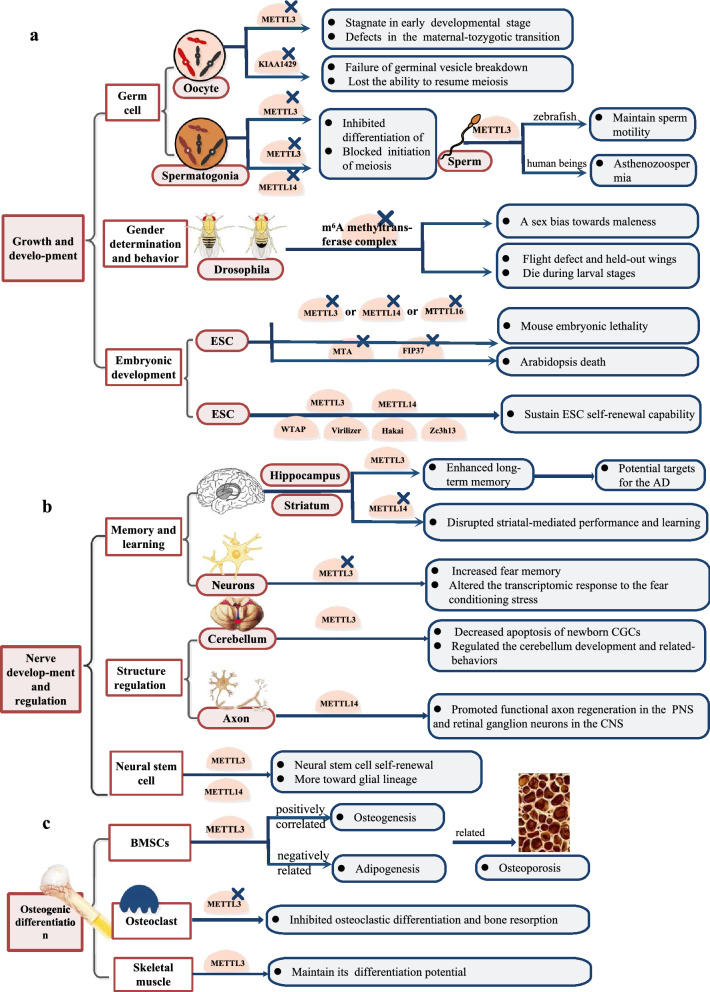
Biological functions of m^6^A methyltransferases in growth and development (**a**), nerve development and regulation (**b**), and osteogenic differentiation (**c**)

M^6^A methyltransferases participate in germ cell maturation and preimplantation embryonic development in animals or plants and even in the reproduction of microorganisms [16]. Although mutations in METTL3 are lethal to embryos, limited research has been conducted on METTL3 in germ cells and more is needed [17, 18], with scientists developing special methods to perform experiments. In female animal germ cells, m^6^A methyltransferases promoted oocyte development and meiosis. In murine and zebrafish oocytes, METTL3 mutations led to arrest in early developmental stage, suppressed maturation and caused defects in the maternal-to-zygotic transition [19, 20] (Fig. 1a). Another study found that KIAA1429 specific deficiency in oocytes led to failure of germinal vesicle breakdown (GVBD), and consequently, the ability to resume meiosis was lost [21] (Fig. 1a). Studying male animal germ cells, Xu K et al. also found that the ablation of METTL3 inhibited the differentiation of spermatogonia and blocked the initiation of meiosis [22] (Fig. 1a); however, Lin et al. suggested that only the combined deletion of METTL3 and METTL14 produced this effect [23, 24] (Fig. 1a). Interestingly, while METTL3 exerted its role in maintaining sperm motility in zebrafish, it caused asthenozoospermia in humans [20, 25] (Fig. 1a). In addition, inactivity of the core RNA methyltransferase (MIS) complex in yeast comprising Ime4 (an orthologue of METTL3), Mum2 (an orthologue of WTAP), and a third ancillary factor, Slz1, inhibited meiosis and sporulation [26, 27] (Fig. 1a). From a micro level, knocking down METTL3 or RBM15 was proven to impair XIST-mediated transcriptional silencing of genes on the X chromosome [28] (Fig. 1a). In addition, m^6^A methyltransferases directly influence sex determination through selective splicing of specific genes in germ cells. Corresponding to METTL3, METTL4, WTAP, KIAA1429, Rbm15/15B and Zc3h13 in mammals, Ime4, dMETTL14, Virm, Fl(2)d, Nito and Flacc orthologues, respectively, together with a newly discovered unique conservative component, xio, form a functional methyltransferase complex that facilitates Sxl pre-mRNA splicing in *Drosophila*, suggesting that this complex has a role in sex determination [29, 30] (Fig. 1a). Mutants show a sex bias towards maleness because knocking down these methyltransferases suppresses male-specific lethal 2 (msl-2), preventing female dosage compensation [14, 31] (Fig. 1a). These *Drosophila* also show flight defects and held out wings. However, fl(2)d, vir, and nito mutants die during larval stages, preventing the analysis of their adult phenotypes [30] (Fig. 1a).

A large body of evidence shows that methyltransferase mutations are embryonic lethal in many species. In mice, knocking out either METTL3 or METTL14 in embryonic stem cells (ESCs) causes inadequate termination of their naive cell state and resistance to differentiation, which accounts for early embryonic lethality [17, 32] (Fig. 1a). METTL16 also exerts its role in the early development of blastocysts [33] (Fig. 1a). In plants, inactivation of Arabidopsis MTA and FIP37, which are orthologues of METTL3 and WTAP in mammals, causes defects in shoot meristems, emergence of lateral roots, and eventually the death of the plant [18, 34, 35] (Fig. 1a). In addition, destabilized METTL3 and METTL14 mRNA encoding developmental regulators in embryonic stem cells help sustain their self-renewal capability [36, 37] (Fig. 1a). Other scientists studied this function of METTL3 to control pluripotency by targeting the SOCS3/JAK2/STAT3 pathway [38] (Fig. 1a). Similarly, depletion of other regulatory subunits, such as WTAP, Virilizer, Hakai and Zc3h13, also impairs ESC self-renewal and induced premature ESC differentiation [39] (Fig. 1a). Notably, increasing the expression of methyltransferase can promote SC reprogramming [40] (Fig. 1a).

### Nerve development and regulation

Memory and learning processes are indispensable to m^6^A methyltransferase. A series of studies found that METTL3 enhances hippocampal long-term memory by promoting the translation efficacy of activity-induced immediate early genes (IEGs), which are DNA binding proteins (e.g., c-Fos, Egr1 and Npas4) that can activate downstream neurotrophic factors to modulate synaptic plasticity and are thus closely related to impaired learning ability and memory formation [41]. Zhang et al. [41] speculated that medicines enhancing METTL3 expression or m^6^A formation may improve learning ability and slow ageing- and/or disease-related memory loss (Fig. 1b). Another study found that METTL14 is essential for the transcriptional regulation of striatal function and learning epitopes. Conditional deletion of METTL14 in striatonigral and striatopallidal neurons increased neuronal excitability, decreased spike frequency adaptation, altered dopamine signalling and seriously disrupted striatal-mediated performance and learning [42] (Fig. 1b). In addition, METTL3 knockdown in adult neurons not only increased fear memory but also altered the transcriptomic response to fear conditioning stress via the regulation of several genes crucial for neuronal systems, such as neurotransmitter receptors, transporters and transcription factors [43] (Fig. 1b). The expression of METTL3 in the cortex and the hippocampus of mice models of Alzheimer’s disease (AD) was significantly higher, which suggests that methylases may be potential targets for the treatment of AD [44].

The regulation of structure and function also includes cerebellar development and axonal regeneration. Some studies have shown precise spatiotemporal expression of the m^6^A methyltransferase METTL3 and a decreased apoptosis rate of new cerebellar granule cells (CGCs) [45] (Fig. 1b), thus maintaining normal Purkinje cell numbers, laminal structure, and the function of glial cell fibres to regulate the development and related behaviours of the mouse cerebellum [46] (Fig. 1b). Moreover, METTL14 is required for promoting injury-induced protein synthesis, functional axon regeneration in the peripheral nervous system and retinal ganglion neurons in the central nervous system of adult mammals [47] (Fig. 1b).

From the perspective of neural stem cell research, some studies have revealed key roles for m^6^A methyltransferases in neural stem cells (NSCs). Lack of METTL14 conspicuously decreased the proliferation and induced premature differentiation of NSCs in vitro, suggesting that METTL14 enhances NSC self-renewal, ensuring the reserves of the neural stem cell bank, while analysis in vivo during cortical neurogenesis showed that a decrease in NSCs in the ventricular zone (radial glial cells, RGCs) was accompanied by fewer cortical neurons [36] (Fig. 1b). Silencing METTL3 induced more NSCs differentiated into the glial lineage, and inhibited morphological maturation of new neurons [48] (Fig. 1b). METTL3 and METTL14 regulate the cell cycle and maintain neural stem cells to enhance the transcriptional coordination of mammalian cortical neurogenesis [49].

### Osteogenic differentiation

METTL3 efficiently and specifically regulates dynamic equilibrium to advance the differentiation of adipocytes and osteoblasts in bone marrow stem cells (BMSCs) [50]. On the one hand, METTL3 expression positively correlates with BMSC-driven osteogenesis. Conditional deletion of METTL3 in BMSCs resulted in incompetent osteogenic differentiation potential, reduced bone mass and impaired bone formation [51, 52] (Fig. 1c). METTL3 knockdown decreased the expression of bone formation-related genes (such as Runx2 and Osterix), precursor (pre-) miR-320 and the PI3K-Akt pathway. Furthermore, the activity of alkaline phosphatase (ALP), the formation of mineralized nodules, the expression of Vegfa and its splice variants vegfa-164 and vegfa-188 were also influenced [52, 53] (Fig. 1c). On the other hand, METTL3 expression is negatively related to BMSC-driven adipogenesis. Loss of METTL3 in BMSCs increased adipogenic differentiation and led to high marrow adiposity [51] (Fig. 1c). Another study explained that METTL3 negatively regulates BMSC adipogenic differentiation. Knocking down METTL3 increased JAK1 protein expression in the JAK1/STAT5/C/EBPb pathway in an m^6^A-YTHDF2-dependent manner, subsequently mediating adipogenic differentiation [54] (Fig. 1c). These pathological changes led to pathological features of osteoporosis in mice. In contrast, the overexpression of METTL3 reduced the probability of oestrogen deficiency-induced osteoporosis [51] (Fig. 1c). Nevertheless, Yu J et al. came to the opposite conclusion. They revealed that METTL3 played an inhibitory role in osteogenesis by inhibiting the calcium deposition and alkaline phosphatase activity of BMSCs and by attenuating the activation of NF-κB, which is universally regarded as a repressor of osteogenesis [55] (Fig. 1c).

To maintain bone homeostasis and preserve skeletal integrity, in addition to osteoblast-mediated bone formation, osteoclast-mediated bone resorption is necessary. METTL3 knockdown inhibited osteoclastic differentiation and bone resorption through an integrated mechanism, including decreasing the expression levels of transcription factors (such as c-Fos and Nfatc1) involved in osteoclast differentiation and factors in bone-resorbing activity (Acp5 and Ctsk), upregulating the expression of the cellular fusion-specific gene Atp6v0d2, entrapping the Traf6 transcript in the nucleus, and subsequently suppressing the activation of MAPK, NF-κB and PI3K-AKT signalling pathways [56] (Fig. 1c). When referring to muscle attached to the bone, METTL3 facilitated mRNA expression of myogenic transcription factor MyoD in proliferative myoblasts to maintain their differentiation potential in skeletal muscle [57] (Fig. 1c).

### Metabolism

**Fig. 2:**
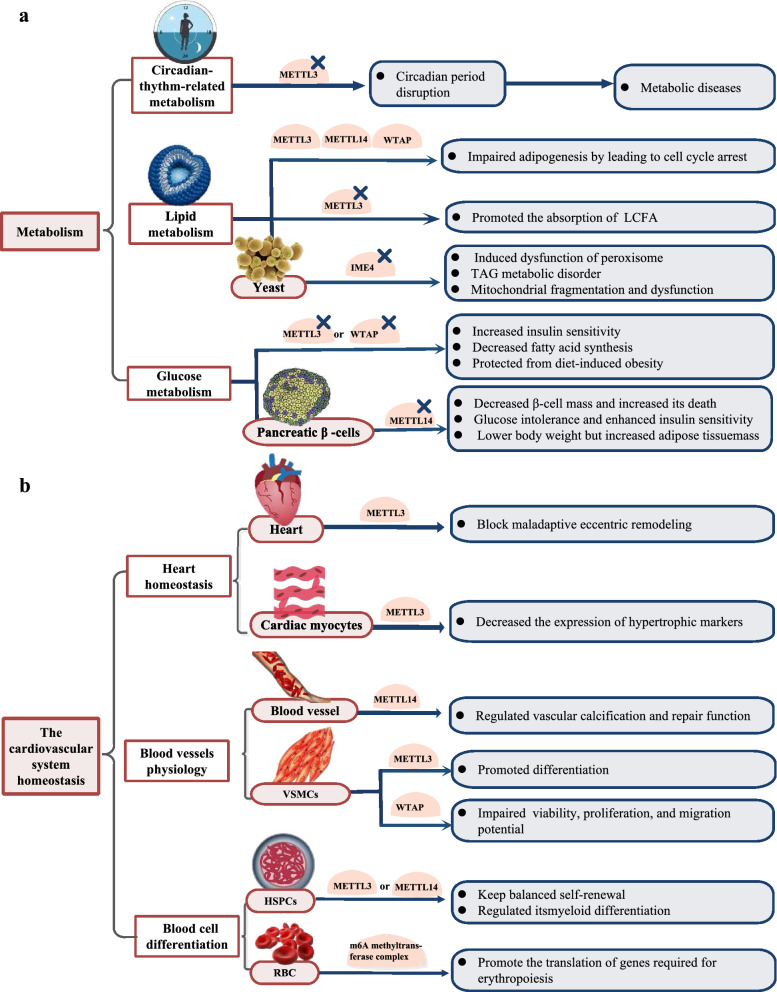
Biological functions of m^6^A methyltransferases in metabolism (**a**) and cardiovascular system homeostasis (**b**)

M^6^A methyltransferases exert their important roles in nutritional physiology and metabolism [58] Among these roles, their ability to regulate circadian rhythm-related metabolism is particularly interesting. Fustin JM et al. found that knocking down METTL3 elicited circadian period elongation by decreasing RNA processing efficiency [59] (Fig. 2a)**.** However, genetic perturbation involving disruption of circadian rhythms can lead to metabolic diseases, especially lipid-related diseases, such as hyperleptinaemia, hypertriglyceridaemia, hepatic steatosis, diabetes, and obesity [60] (Fig. 2a). The absence of liver Bmal1, an essential component of the mammalian circadian rhythm regulatory network, leads to ROS accumulation and disruption of lipid metabolism, but via knockdown of m^6^A methyltransferase METTL3, lipid accumulation can be reduced because of a decrease in peroxisome proliferator-activator a (PPaRa), m^6^A abundance, extended mRNA lifetimes and increased expression [60] (Fig. 2a). Furthermore, m^6^A methyltransferases also act on lipid metabolism independently. Some studies have suggested that m^6^A methyltransferases negatively correlate with adipogenesis [61, 62] (Fig. 2a). WTAP, together with METTL3 and METTL14, impaired adipogenesis by inducing cell cycle arrest during the mitotic clonal expansion (MCE) of adipocytes [62] (Fig. 2a). Mechanistically similar to its depletion in osteoclasts, depletion of METTL3 entrapped Traf6 transcripts in the nucleus and suppressed the NF-kB and MAPK signalling pathways of inflammation, promoting the absorption of long-chain fatty acids (LCFAs) [63] (Fig. 2a). A similar effect was found in the metabolism of fungi. IME4 (an orthologue of METTL3) deletion induced dysfunction of peroxisomes, which are the sole sites of fatty acid β-oxidation in yeast [64] (Fig. 2a). In addition to its direct effect on regulating triacylglycerol (TAG) metabolism in haploid cells, IME4 deletion caused mitochondrial fragmentation and dysfunction, which indirectly influenced TAG metabolism [65] (Fig. 2a).

In addition, carbohydrate metabolism is involved. The relationship between m^6^A methyltransferase and glucose metabolism was mentioned above. Because diabetes is a prevalent metabolic disease, many studies on methyltransferases in diabetes have been performed in recent years. Reduced METTL3 in mice increased insulin sensitivity, decreased fatty acid synthesis and protected the mice from diet-induced obesity [66] (Fig. 2a). A similar conclusion on lipid and glucose metabolism had been reached in other studies of WTAP and METTL3 [62, 67] (Fig. 2a). In addition, by changing the stability of ICAM-1 mRNA, METTL3 knockdown repressed the apoptosis of human lens epithelial cells (HLECs) in diabetic cataracts caused by high levels of glucose [68] (Fig. 2a). Compared to these methyltransferase-disease interactions, the relationship of METTL14 with pancreatic β cells seems to be the closest. METTL14 deficiency destroys pancreatic β-cell homeostasis, specifically shown as decreased β-cell mass and increased cell death, as well as glucose intolerance, enhanced insulin sensitivity, and reduced body weight despite increased adipose tissue mass [69]. Other studies that reached similar conclusions explained that METTL14 regulates the functions of β cells through the insulin/IGF1-AKT-PDX1 or IRE1a/sXBP-1 pathway [70, 71] (Fig. 2a).

Knockdown of METTL3/14 inhibited the expression levels and activities of the drug metabolizing enzyme cytochrome P450 in HepaRG and Huh-7 cells [72].

### Cardiovascular system homeostasis

The cardiovascular system consists of the heart and blood vessels. Recently, some studies have tried to explain the relationship between m^6^A methyltransferases and the growth of cardiomyocytes. However, interestingly, because of differences in study designs and modelling methods, these studies came to opposite conclusions. Dorn et al. reported that METTL3 maintained cardiac homeostasis and the heart response to pressure-overload stress. Increasing the expression of the m^6^A methyltransferase METTL3 in the heart drove spontaneous, compensated hypertrophy but did not affect cardiac function, whereas METTL3 knockdown led to morphological and functional signs of heart failure, which demonstrated that METTL3 may have the ability to block maladaptive eccentric remodelling [73] (Fig. 2b). In contrast, Kmietczyk V et al. demonstrated that METTL3 conspicuously decreased the expression of hypertrophic markers Nppa and Nppb to prevent pathological growth in cardiac myocytes [74] (Fig. 2b). METTL3 downregulated the expression levels of transcription factor EB (TFEB), which is directly involved with lysosomal biogenesis and autophagy, subsequently inhibiting autophagy and increasing the apoptosis rate of H/R-treated cardiomyocytes [75] (Fig. 2b).

Blood vessel physiology is complex and involves multiple molecules in multiple cells. METTL14 selectively hypermethylates the transcript of Klotho, a vascular system-protecting protein, promoting its degradation and attenuating the harmful expression of this protein induced by indoxyl sulfate in vascular calcification, thereby decreasing the vascular repair function. Interestingly, the forced expression of METTL14 in stressed human artery smooth muscle cells (HASMCs) has the opposite effect, which may predict the therapeutic potential of METTL14 in vascular calcification-involved diseases [76] (Fig. 2b). However, METTL14 seems to be associated with inflammatory infiltrates and neovascularization to lead to a greater risk of human abdominal aortic aneurysm (AAA) rupture [77] (Fig. 2b). One study suggested that silencing METTLE3 not only reduced the expression of VSMC-specific markers, including α-SMA, SM22α, calponin, and SM-MHC but also decreased the expression of paracrine factors, including VEGF, HGF, TGF-β, GM-CSF, bFGF, and SDF-1, which revealed a positive role for METTL3 in vascular smooth muscle differentiation [78] (Fig. 2b). Another study reported that WTAP, a component of m^6^A methyltransferases, exerted a negative role by impairing the viability, proliferation, and migration potential of vascular smooth muscle cells (VSMCs) by mechanistically regulating p16 via m^6^A modification, thereby preventing arterial restenosis induced by intimal hyperplasia [79] (Fig. 2b).

Moreover, m^6^A methyltransferases exert their role in the generation and differentiation of blood cells. METTL3 was reported to maintain balanced self-renewal and differentiation in the fate determination of haematopoietic stem/progenitor cells (HSPCs) [80] (Fig. 2b). It can repress arterial-endothelial Notch activity, thereby promoting HSPC generation through the endothelial-to-haematopoietic transition (EHT) [80, 81] (Fig. 2b). In vitro knockdown of METTL3 or METTL14 in HSPCs led to myeloid differentiation [82, 83] (Fig. 2b), whereas in vitro deletion blocked HSC differentiation to cause an accumulation of HSCs in the bone marrow and a reduction in reconstitution potential [84] (Fig. 2b), which can be interpreted as regulating the expression of the asymmetric or symmetric cell division marker MYC in HSPCs [85]. Moreover, the m^6^A methyltransferase complex promoted the translation of genes required for human erythropoiesis, including those encoding SETD histone methyltransferases, ribosomal components, and poly(A) RNA-binding proteins [86].

### Infection and immunity

**Fig. 3:**
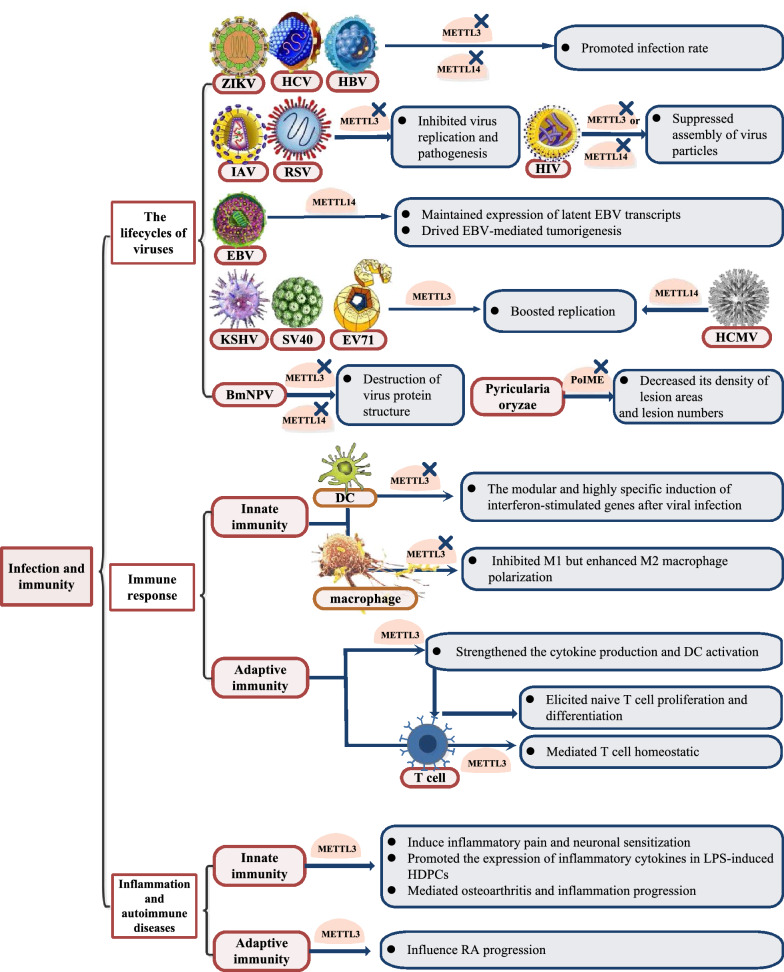
Biological functions of m^6^A methyltransferases in infection and immunity

Recent studies suggest that m^6^A methyltransferases play diverse roles in either restricting or modulating the lifecycles of viruses. We first focus on RNA viruses, which are classified into positive-sense, single-stranded RNA viruses, such as Flaviviridae. Zika virus (ZIKV) replication efficiency was enhanced after METTL3 or METTL14 knockdown, modifying its host mRNA landscape [87] (Fig. 3), whereas the hepatitis C virus (HCV) infection rate was increased, not through viral RNA replication but through increased production of infectious viral particles [88] (Fig. 3). However, in another single-stranded RNA virus that similarly replicates in the cytoplasm, enterovirus 71 (EV71) of the Picornaviridae family displayed the opposite pattern. METTL3 increased SUMOylation and ubiquitination of the viral RNA polymerase 3D to boost viral replication, and through interaction, 3D recruits METTL3 to sites of viral RNA replication [89] (Fig. 3). In influenza A virus (IAV) and respiratory syncytial virus (RSV), for which infection by either is characterized by respiratory symptoms, inactivation of METTL3 inhibited virus replication and pathogenesis [90, 91] (Fig. 3). In addition to suppressing viral replication as explained above, by reducing Rev protein, which preferentially interacts with methylated RRE, the export of viral RNA is constrained [92] (Fig. 3), and silencing of METTL3 or METTL14 decreased HIV-1 Gag expression, which is crucial in the assembly of virus particles [93, 94] (Fig. 3). In regard to DNA viruses, METTL14 not only maintains the expression of latent Epstein-Barr virus (EBV) transcripts but also drives EBV-mediated tumorigenesis via direct interaction with the viral-encoded latent oncoprotein EBNA3C [95] (Fig. 3). METTL14 also plays a positive role in the growth cycle of human cytomegalovirus (HCMV), without which interferon β accumulates to reduce virus protein expression and reproduction [96] (Fig. 3). Consistent with the role of m^6^A methyltransferases in the DNA virus infection, in Kaposi’s sarcoma-associated herpesvirus (KSHV) and simian virus 40 (SV40), METTL3 reduced the post-transcriptional accumulation of the major viral lytic transactivator ORF50 [97–99] (Fig. 3) and enhanced the translation of late SV40 transcripts, respectively [100] (Fig. 3). Another DNA virus, hepatitis B virus (HBV), is an exception. METTL3 and METTL14 depletion led to increased expression of the HBc and HBs proteins, consequently promoting the progression of infection [101] (Fig. 3). This regulatory effect of methyltransferases in pathogenic infections is found not only in humans but also in animals and plants. Silencing of METTL3 or METTL14 decreased the *Bombyx mori nucleopolyhedrovirus* (BmNPV) structural protein VP39 [102] (Fig. 3). Moreover, Pyricularia oryzae, a filamentous phytopathogenic fungus that causes unfavourable declines in rice production, showed decreased density at lesion areas and lesion numbers after deletion of PoIME (an m^6^A methyltransferase in *P. oryzae*) [103] (Fig. 3).

In addition to acting directly on viruses, m^6^A methyltransferases can regulate the immune response to infection [104] (Fig. 3). In antiviral innate immunity, METTL3 depletion results in the modular and highly specific induction of hundreds of interferon-stimulated genes after viral infection, constituting one of the first lines of pathogen defence [105] (Fig. 3). However, in macrophage polarization, METTL3 plays a dual regulatory role, and knocking down METTL3 significantly inhibits M1 macrophage polarization, which has high bactericidal and proinflammatory activities, but enhances M2 macrophage polarization, which has anti-inflammatory properties [106] (Fig. 3). METTL3 also enhanced the translation of CD40, CD80 and cytokine IL-12 transcripts in dendritic cells, strengthening the cytokine production induced by TLR4/NF-κB signalling and dendritic cell (DC) activation, further eliciting the proliferation and differentiation naive T cells, which are involved adaptive immunity [107] (Fig. 3). METTL3 induced the decay of SOCS family genes that encode STAT signalling inhibitory proteins, consequently promoting IL-7-mediated STAT5 activation and T cell homeostatic proliferation and differentiation [108] (Fig. 3). Consistent with these observations, in CD4^+^ regulatory T cells (Tregs), SOCS targets the IL-2-STAT5 signalling pathway to sustain the suppressive functions of Tregs [109] (Fig. 3).

Recent work on inflammation has yielded some intriguing mechanistic insights into how it might be affected by m^6^A methyltransferases. METTL3 fostered pri-miR-65-3p processing in a microprocessor protein DiGeorge critical region 8-dependent manner to induce inflammatory pain and neuronal sensitization [110]. It also suppressed the expression of MyD88S, which inhibited inflammatory cytokine production, and then promoted the expression of inflammatory cytokines in LPS-induced human dental pulp cells (HDPCs), as well as related markers in the NF-κB and MAPK signalling pathways [111]. Corresponding with this signalling pathway, NF-κB signalling, together with extracellular matrix ECM synthesis, is involved in mediating progression of METTL3 in osteoarthritis [112], whereas another study demonstrated that, in contrast, METTL3 knockdown facilitates LPS-induced inflammation by regulating MAPK signalling [113]. In autoimmune diseases, although the overexpression of METTL3 has been shown to attenuate the inflammatory response induced by LPS in macrophages dependent on NF-κB to influence rheumatoid arthritis (RA) progression [114], the effect of METTL3 on systemic lupus erythaematosus (SLE) remains a matter of speculation [115].

### Tumour progression

Recently, the function of m^6^A methyltransferases in oncology has become a focus, and a variety of studies have led to breakthroughs in understanding and treatment.

**Fig. 4:**
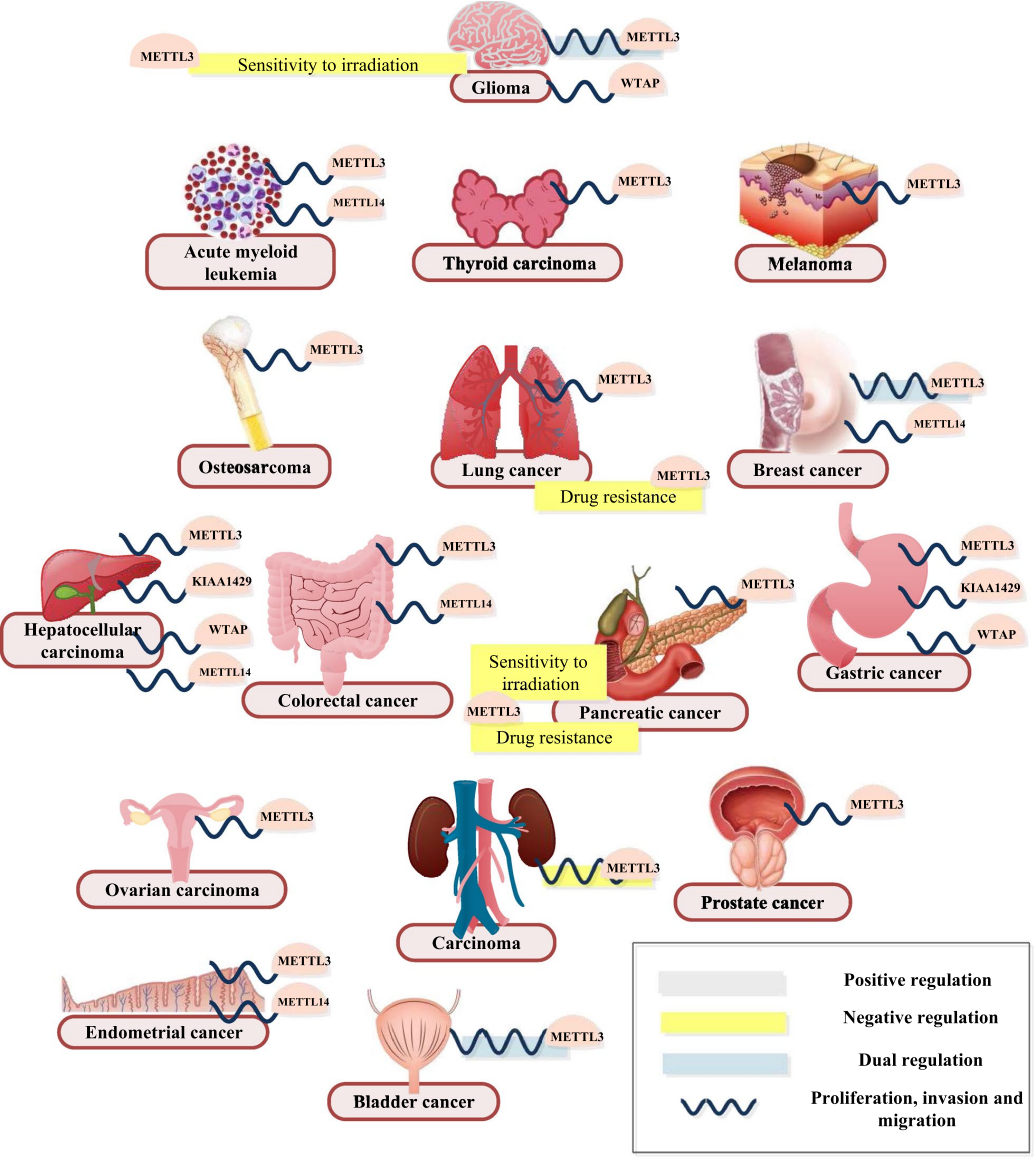
Biological functions of m^6^A methyltransferases in tumour progression

The effects of m^6^A methyltransferases on the proliferation, invasion and metastasis of different tumours vary tremendously. In the majority of tumours of comparatively high incidence, such as digestive system tumours, including gastric cancer (GC) [116–118], colorectal cancer (CRC) [119–121], hepatocellular carcinoma (HCC) [122, 123] and pancreatic cancer (PAAD) [124], as well as lung cancer (LCA) [125, 126], endometrial cancer [127], thyroid carcinoma [128], prostate cancer [129], osteosarcoma [130], melanoma [131], ovarian carcinoma [132] and more (Fig. 4), knocking down METTL3 was verified to inhibit the proliferation, invasion and migration of cancer cells in vitro by regulating the expression of relevant genes and pathways. Some studies even showed METTL3 roles in tumorigenesis- and metastasis-promoting effects in vivo, including in CRC [121], GC [133], HCC [123, 134] and prostate cancer [129] (Fig. 4). Of particular interest, in GC [116, 117], LCA [135] and ovarian carcinoma [132] the epithelial-mesenchymal transition (EMT) control of METTL3 seems to be particularly important. In addition, in acute myeloid leukaemia (AML), METTL3 depletion caused a favourable outcome by delaying the occurrence of disease through the promotion of the terminal myeloid differentiation of HSPCs and impairment of AML cell survival [82] (Fig. 4). In addition to METTL3, other m^6^A methyltransferase components, namely, WTAP and KIAA1429, also play adverse roles in GC [130, 136] and HCC [137, 138] progression, respectively. METTL14 is also expressed in CRC [139], HCC [134], AML [83], breast cancer [140] and endometrial cancer [127] (Fig. 4). Although the effects of METTL3 are mechanistically similar to those in GC and LCA in terms of the EMT and PI3K-Akt-mTOR pathways, in renal cell carcinoma (RCC), knocking down METTL3 significantly promoted cell proliferation, migration and invasion [141] (Fig. 4). Thus, opposite functions of METTL3 are not unique and are similar to those of its counterpart, METTL14, in GC and CRC [142, 143]. Interestingly, in glioma and breast cancer, the role of m^6^A methyltransferases remains controversial. Some studies have suggested that overexpression of METTL3 profoundly inhibited the proliferation, tumorigenicity and migration ability of glioma [144, 145], breast cancer [146] or bladder cancer cells [147] by altering the mRNA expression of genes or proteins. Paradoxically, other studies have shown that by silencing METTL3 [148–153] or WTAP (only in glioma) [154], similar cell growth and aggressiveness inhibition was achieved (Fig. 4).

Because m^6^A methyltransferase plays a vital role in the proliferation, invasion and migration of cancer cells, its level can be used to judge the stage and prognosis of tumours. Survival analysis showed that METTL3 serves as a prognostic factor for poor outcomes for CRC [121, 143], GC [117, 133], pancreatic cancer [124], HCC [122, 123] and thyroid carcinoma [128] patients. Specifically, Hua W et al. pointed out that a high level of METTL3 was significantly associated with tumour TMN grade and FIGO stage [132] (Fig. 4). In contrast, corresponding to the aforementioned functions, RCC patients with high METTL3 expression had an obviously longer survival time [141, 155] (Fig. 4). Moreover, WTAP expression served as an independent predictor for the survival of patients with HCC [137], GC [136], RCC [156] and high-grade serous ovarian carcinoma [157]. A high WTAP level is also closely correlated with increased postoperative recurrence risk of bladder cancer [158] and glioma grade [159] (Fig. 4). However, METTL14 acted in the opposite manner in HCC [134], CRC [143] and GC [142]. In other words, METTL14 downregulation demonstrated adverse clinical outcomes (Fig. 4).

In terms of treatment, while METTL3-silenced GSCs and pancreatic cancer cells showed enhanced sensitivity to irradiation [160, 161], METTL3-depleted cells induced NSCLC and pancreatic cancer chemotherapeutic drug resistance to gemcitabine, 5-fluorouracil, cisplatin, etc. [161, 162] (Fig. 4).

## Conclusions

M^6^A is acknowledged as one of the most ubiquitous and abundant mRNA methylation modifications in eukaryotes. Therefore, m^6^A methyltransferases have attracted increasing attention due to their various functions in mediating growth and development, metabolism, behavioural activity and even disease development. In addition to these major aspects, their effects in other areas have been discovered, including drug toxicity [163] and cytoplasmic turnover [164]. In this emerging and hot research direction, many gratifying results have been revealed in recent years, among which some difficult miscellaneous diseases have been effectively resolved with current medical level advancements. These achievements may provide new approaches for delaying memory decline in AD, interrupting metastasis and recurrence of tumours, and controlling autoimmune disease progression. Nevertheless, there remains many functions in related fields that deserve further exploration.

According to the current research foundation, the following points need to be addressed. First, as far as m^6^A methyltransferases are concerned, existing research on several major components (METTL3/METTL14/WTAP) has been relatively intensive, but studies on the later-discovered regulatory subunits, such as METTL16 [15], METTL5, RBM15, VIRMA, and ZCCHC4 [165], are few and cursory. Secondly, there is also room for exploration into the interactions between components including m^6^A methyltransferases, demethylases and binding proteins. With the increasing number of discovered methyltransferases and binding proteins, it still unclear if any special m^6^A modification sites mediated by different methyltransferases; if any special m^6^A modification sites recognized by different binding proteins; if any special interactions among methyltransferases, demethylases and binding proteins. For instance, In addition to the basic structure and functional relationships mentioned in the introduction, Sorci M et al. illustrated multiple dimensions of mutual regulation in mRNA translation and stability, by which either knockdown or overexpression of METTL3 upregulated WTAP protein and influenced its homeostasis. In addition, WTAP upregulation can have a carcinogenic effect only in the presence of a functional m^6^A methylation complex [166]; that is, its function is m^6^A-dependent. Most m^6^A methyltransferases act as indispensable writers of N6-methyladenosine to play roles in biology. However, Qian JY et al. reported that KIAA1429 can change CDK1 transcript stability and extend its half-life to induce breast cancer [167]. Lin S et al. revealed that METTL3 facilitated the translation of the mRNAs of epidermal growth factor receptor (EGFR) and the Hippo pathway effector TAZ to promote the progression of human lung cancer [125], which suggests that some aspects of m^6^A methyltransferase can regulate life processes in an m^6^A-independent manner. This possibility has attracted little attention but may eventually lead to improvements in this field. Thirdly, the bio-function of m^6^A methyltransferase remains contradictory. For instance, in osteogenesis, while most scientists have proved that METTL3 expression in BMScs is positively correlated with osteogenesis and negatively correlated with adipogenesis [51, 52], Yu J et al. revealed that METTL3 inhibits the calcium deposition and alkaline phosphatase activity of BMSCs and attenuates the activation of NF-κB to impair osteogenesis [55]. In another example, METTL3 exhibited an inhibitory role in the proliferation, tumorigenicity and migration ability in glioma cells [144], which had a paradoxical effect [149]. Similar but different types of cells used in experiments, dynamic impacts at different stages of the same life process, and horizontal staggering of sequencing-based methodologies used in each study may contribute to the contradictory results obtained. This outcome is a reminder that more accurate detection methods and more extensive cooperation and communication are needed at the international level. Last, but most significant, more exploration is needed for the quantification of modifications on a transcriptome-wide level, identification of precise sites and discovery of upstream and downstream regulation mechanisms of m^6^A methyltransferases. Currently, very few drugs are based on m^6^A methyltransferase function, and some related ideas are at the speculative stage. We anticipate an increased transition from mature in vitro cell experiments to in vivo studies and expansion of the development of some targeted clinical drugs. All these findings may reveal insights for developing novel preventive and curative strategies for related diseases.

## Data Availability

Not applicable.

## Abbreviations

AD: Alzheimer’s disease
ALP: Alkaline phosphatase
BMSCs: Bone marrow stem cells
BmNPV: Bombyx mori nucleopolyhedrovirus
CGSs: Cerebellar granule cells
CNS: Central nervous system
EBV: Epstein-Barr virus
ESC: Embryonic stem cells
EV71: Enterovirus 71
GVBD: Germinal vesicle breakdown
HASMC: Human artery smooth muscle cell
HBV: Hepatitis B virus
HCV: Hepatitis C virus
HCMV: Human cytomegalovirus
HIV: Human immunodeficiency virus
HSPCs: Haematopoietic stem/progenitor cells
IAV: Influenza A virus
IEGs: Immediate early genes
KSHV: Kaposi’s sarcoma-associated herpesvirus
LCFA: Long-chain fatty acids
M^6^A: N6-methyladenosine
METTL3: Methyltransferase-like 3 protein
METTL14: Methyltransferase-like 14 protein
NSCs: Neural stem cells
PNS: Peripheral nervous system
PolyA: Polyadenosinic acid
RBC: Red blood cell
SV40: Simian virus 40
TAG: Triacylglycerol
Tregs: CD4 + regulatory T cells
VSMCs: Vascular smooth muscle cell
WTAP: Wilm’s tumour-1-associated protein
ZIKV: Zika virus
